# Supplementary material for: Predicted norovirus resurgence in 2021–2022 due to the relaxation of nonpharmaceutical interventions associated with COVID-19 restrictions in England: a mathematical modeling study
Source: BMC Med. 2021 Nov 9;19:299. doi: 10.1186/s12916-021-02153-8 (PMC8577179; doi:10.1186/s12916-021-02153-8)
Supplement: Supplementary file 5 — Additional file 5: Figure S1. Comparison of simulations and cases reported to SGSS between July 2019 to June 2021. [file 12916_2021_2153_MOESM5_ESM.docx]

**Figure S1**

Figure S1. Comparison of simulations (green lines) and cases reported to SGSS (brown line) between July 2019 to June 2021.
