# Supplementary material for: Predicted norovirus resurgence in 2021–2022 due to the relaxation of nonpharmaceutical interventions associated with COVID-19 restrictions in England: a mathematical modeling study
Source: BMC Med. 2021 Nov 9;19:299. doi: 10.1186/s12916-021-02153-8 (PMC8577179; doi:10.1186/s12916-021-02153-8)
Supplement: Supplementary file 6 — Additional file 6: Figure S2. Investigating the impact of reduced contact patterns from July 2021 compared to pre-pandemic. [file 12916_2021_2153_MOESM6_ESM.docx]

**Figure S2**

**Investigating the impact of reduced contact patterns from July 2021 compared to pre-pandemic**

**Figure S2. Estimates of the impact of changing contact patterns due to COVID-19 restrictions on norovirus A) incidence and B) susceptibility to symptomatic infection from January 2019 to June 2023.** In each panel each colour represents simulations assuming a duration of asymptomatic infectiousness of 15 (light red) or 20 (red) days, and allowing for different assumptions about under-reporting of norovirus incidence within Harris et al. [15]; solid lines assume no under-reporting and dashed lines assume 20% underreporting. UP: under-reporting, sim: simulated duration of asymptomatic infectiousness in days.
